# CYP2D6 gene polymorphism and apatinib affect the metabolic profile of fluvoxamine

**DOI:** 10.3389/fphar.2022.985159

**Published:** 2022-09-02

**Authors:** Zhize Ye, Bingbing Chen, Nanyong Gao, Qihui Kong, Xiaoqin Hu, Zhongqiu Lu, Jianchang Qian, Guoxin Hu, Jianping Cai, Bin Wu

**Affiliations:** ^1^ Institute of Molecular Toxicology and Pharmacology, School of Pharmaceutical Sciences, Wenzhou Medical University, Wenzhou, China; ^2^ Emergency Center, The First Affiliated Hospital of Wenzhou Medical University, Wenzhou, China

**Keywords:** CYP2D6, fluvoxamine, apatinib, interaction, metabolism

## Abstract

This study aimed 1) to investigate the influence of CYP2D6 variants on the catalyzing of fluvoxamine, and 2) to study the interaction between fluvoxamine and apatinib. An enzymatic reaction system was setup and the kinetic profile of CYP2D6 in metabolizing fluvoxamine was determined. *In vivo*, drug-drug interaction was investigated using Sprague–Dawley (SD) rats. Fluvoxamine was given gavage with or without apatinib. Ultra-performance liquid chromatography-tandem mass spectrometry (UPLC-MS/MS) was used to determine the concentrations of fluvoxamine and desmethyl-fluvoxamine. The results demonstrated that the relative clearance rates of CYP2D6.A5V, V104A, D337G, F164L, V342M, R440C and R497C increased significantly compared with CYP2D6.1, ranging from 153.626% ± 6.718% to 394.310% ± 33.268%. The activities of other variants reduced to different extent, or even lost function, but there was no statistical difference. The IC_50_ of apatinib against fluvoxamine disposition was determined, which is 0.190 μM in RLM and 6.419 μM in HLM, respectively. *In vivo*, apatinib can enhance the plasma exposure of fluvoxamine remarkably characterized by increased AUC, Tmax and Cmax. Meanwhile, the produce of desmethyl fluvoxamine was dramatically inhibited, both AUC and C_max_ decreased significantly. Mechanistically, apatinib inhibit the generation of fluvoxamine metabolite with a mixed manner both in RLM and HLM. Furthermore, there were differences in the potency of apatinib in suppressing fluvoxamine metabolism among CYP2D6.1, 2 and 10. In conclusion, CYP2D6 gene polymorphisms and drug-drug interaction can remarkably affect the plasma exposure of fluvoxamine. The present study provides basis data for guiding individual application of fluvoxamine.

## Introduction

Fluvoxamine is commonly prescribed to treat depression and anxiety disorders with mechanism of selective inhibit serotonin reuptake (Yuan et al., 2020). Central nervous system symptoms, rash, gastrointestinal symptoms, and suicidal tendencies are common adverse reactions during medicine of fluvoxamine (Lenze et al., 2020). Its clinical efficacy is highly variable among individuals. Drug efficacy stratification may be the result of individual differences in blood concentrations which caused by genetic polymorphisms of metabolic enzymes and drug interactions. Cytochrome P450 family member 2D6 (CYP2D6) is a major enzyme involved in catalyzing metabolism of fluvoxamine, which produced desmethyl fluvoxamine (Hicks et al., 2015; Zastrozhin et al., 2021). However, there are genetic polymorphism of CYP2D6 resulting in large inter-individual variability in enzyme activity, further leading to subtherapeutic phenomena or severe adverse effects (Zastrozhin et al., 2021). Moreover, interactions between fluvoxamine and other drugs are frequently being reported. A study found one fatality in a woman who was taking clotiapine, 7-aminoclonazepam, propranolol, gabapentin and haloperidol alongside fluvoxamine developing antipsychotic malignant syndrome (Vignali et al., 2021). Interactions of fluvoxamine with antiepileptic or antidepressant drugs have also been issued (Mula and Trimble, 2003; Spina et al., 2016). Therefore, defining the correlation between CYP2D6 genotype and fluvoxamine metabolic phenotype, identifying drugs that could potential interact with fluvoxamine are helpful for personalized medicine.

Apatinib is a small molecule drug that targeting inhibition of angiogenesis (Wang N. et al., 2020). It’s approved to be safe and effective after failure of standard chemotherapy in advanced gastric cancer (Geng et al., 2018). Interestingly, it is worth noting that apatinib is a pan CYP inhibitor (Zhou et al., 2014). Therefore, the research on the interaction between apatinib and other CYP substrate drugs has gradually attracted people’s attention. Statistics data display that cancer patients usually complicated with various psychological symptoms, especially depression and anxiety (Wang YH. et al., 2020). This will diminish benefits of medicine and affects the quality life of patients. Therefore, the combined application of apatinib and fluvoxamine is a feasible clinical treatment option. However, the interaction between them has not been unveiled.

Herein, we evaluated the catalytic activity of CYP2D6.1 and other 23 variants on the disposition of fluvoxamine. In addition, we used microsomes and Spragge-Dawley (SD) rats to clarify the interaction between fluvoxamine and apatinib *in vitro* and *in vivo*. The results will provide fundamental data to facilitate the precision medicine application of fluvoxamine.

## Materials and methods

### Chemicals and reagents

Fluvoxamine maleate was bought from Shanghai Canspec Scientific & Technology Co., Ltd. Desmethyl fluvoxamine was obtained from TRC Ltd. (Toronto, Canada). Diazepam was purchased from Shanghai Xudong Haipu Pharmaceutical Co., Ltd. and used as internal standard. Apatinib was obtained from Beijing Sunflower Technology Development Co., Ltd. Sodium carboxymethyl cellulose (CMC-Na), methanol, acetonitrile (ACN) and formic acid were purchased from Merck (Darmstadt, Germany). Microsomes were purchased from Corning Life Sciences Co., Ltd. CYP2D6 and cytochrome B5 were prepared as previously issued (Cai et al., 2016).

### UPLC-MS/MS and condition

A newly developed and validated UPLC-MS/MS method was used to detect fluvoxamine and desmethyl fluvoxamine. The analytes were separated on a BEH C18 column (2.1 × 100 mm, 1.7 μm; Waters Corp., Millipore, Bedford, MA, United States), which incubated at 40°C. The mobile phase was consisted of 0.1% formic acid and ACN, and elution at 0.40 ml/min for 3.0 min with a gradient condition. The program was set as 10%–90% ACN (0–1.0 min), 90%–10% ACN (1.0–2.0 min), and 10% ACN (2.1–3.0 min).

### Determine enzymatic kinetic parameters of recombinant human CYP2D6 using fluvoxamine

Incubation system was dissolved in phosphate buffered saline which contained 1 pmol CYP2D6.1 or variants, 50 μg/ml cytochrome B5, 0.5–50 μM fluvoxamine. Before the reaction, the mixture was pre-incubated at 37°C for 5 min. Subsequently, add 1 mM nicotinamide adenine dinucleotide phosphate oxidase to initiate the reaction. 20 min’ later, the reaction was terminated. Add acetonitrile twice volume as much as reaction system and 20 μl internal standard to the mixture. After vortexing and centrifugation, the supernatant was taken and subjected to UPLC-MS/MS.

### Animal experiments

Animal ethics was reviewed and approved by Wenzhou Medical University. Male rats weighed 180–220 g were supplied by Vital River Laboratories (Beijing, China), and adaptive feeding for a week. SD rats were divided into two groups. Group A served as control, dosing of vehicle (0.5% CMC-Na). Group B was administrated 40 mg/kg apatinib. 30 min’ later, fluvoxamine (10 mg/kg) was given orally to the rats. Then, the vein blood was collected at 0, 0.25, 0.5, 1, 2, 3, 4, 5, 6, 8, 10, 12, and 24 h after administration. The sample was prepared and subjected to UPLC-MS/MS examination.

### Microsomes incubation assay

The microsomes reaction system was set up as indicated in above. In briefly, the reaction was carried out in PBS. The buffer was consisted of 0.2 mg/ml RLM or HLM, 0.5–50 μM fluvoxamine. NADPH was used to initiate the reaction. To determine the half maximal inhibitory concentration (IC_50_), the concentration of apatinib was set at 0.01, 0.1, 1, 10, 25, 50, and 100 μM. To determine the mechanism underlied the inhibition, the concentration of fluvoxamine was set according to the K_m_ value, while the concentration of apatinib was set at 0, 0.25, 0.5, 1 μM according to the IC_50_ as well. After incubation, the samples were prepared and determined by UPLC-MS/MS.

### Statistical analysis

Lineweaver-Burk double reciprocal plot was performed on GraphPad Prism 5.0 software. The kinetic parameters were obtained using non-compartmental model fitting by Drug and statistics (DAS) software 3.0. The corresponding drug-time curves were drawn by Origin 8.0. All data are expressed as Mean ± SD. Statistical analysis was performed by independent samples *t*-test using GraphPad Prism 5.0 software. *p* < 0.05 indicates a significant difference.

## Results

### Development of UPLC-MS/MS assay to determine fluvoxamine and desmethyl fluvoxamine

**FIGURE 1:**
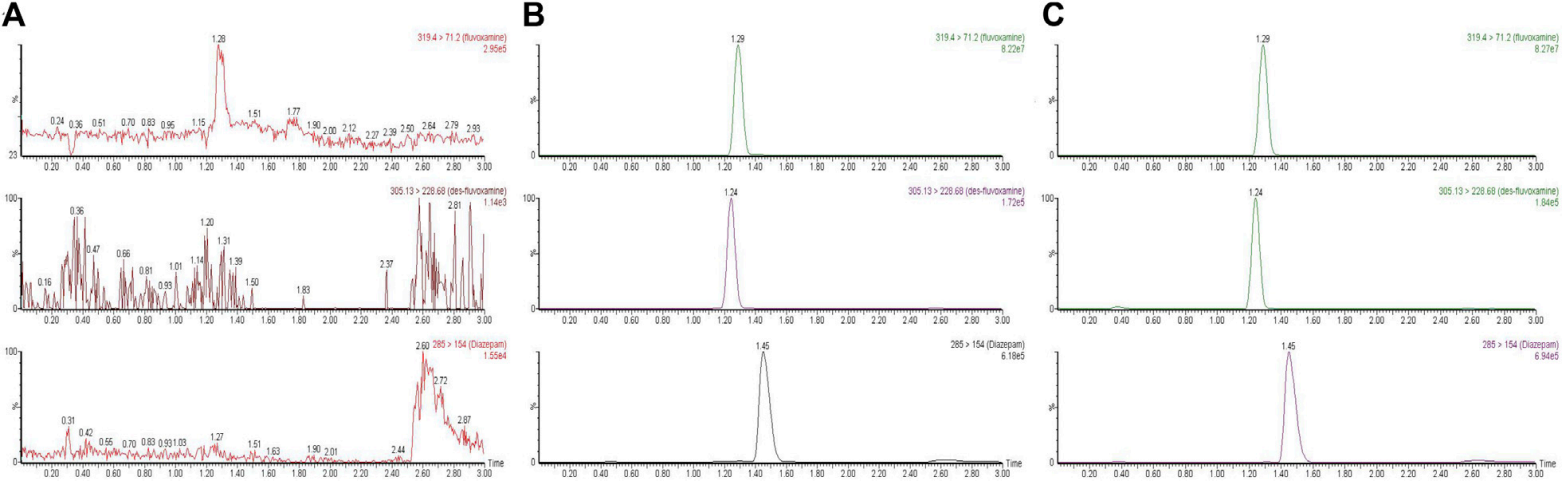
Representative chromatograms of fluvoxamine, desmethyl fluvoxamine and IS. **(A)** a blank plasma sample. **(B)** A blank plasma sample spiked with fluvoxamine, desmethyl Fluvoxamine and IS. **(C)** Rat’s plasma sample after dosing.

To detect the analytes, a UPLC-MS/MS method was developed and validated. The linear range, precision, accuracy, recovery, matrix effect and stability were evaluated. The detail data was presented in the Supplementary Information. In briefly, the monitoring transitions of diazepam, fluvoxamine and desmethyl fluvoxamine were m/z 285→ 154, m/z 319.4→ 71.2 and m/z 305.13→ 228.68, respectively. As Figure 1 showed, there was no obvious endogenous interference. The retention time of diazepam, fluvoxamine and desmethyl fluvoxamine were 1.45, 1.29 and 1.24 min, accordingly.

### Kinetic characterization of recombinant human CYP2D6 in catalyzing of fluvoxamine

**FIGURE 2:**
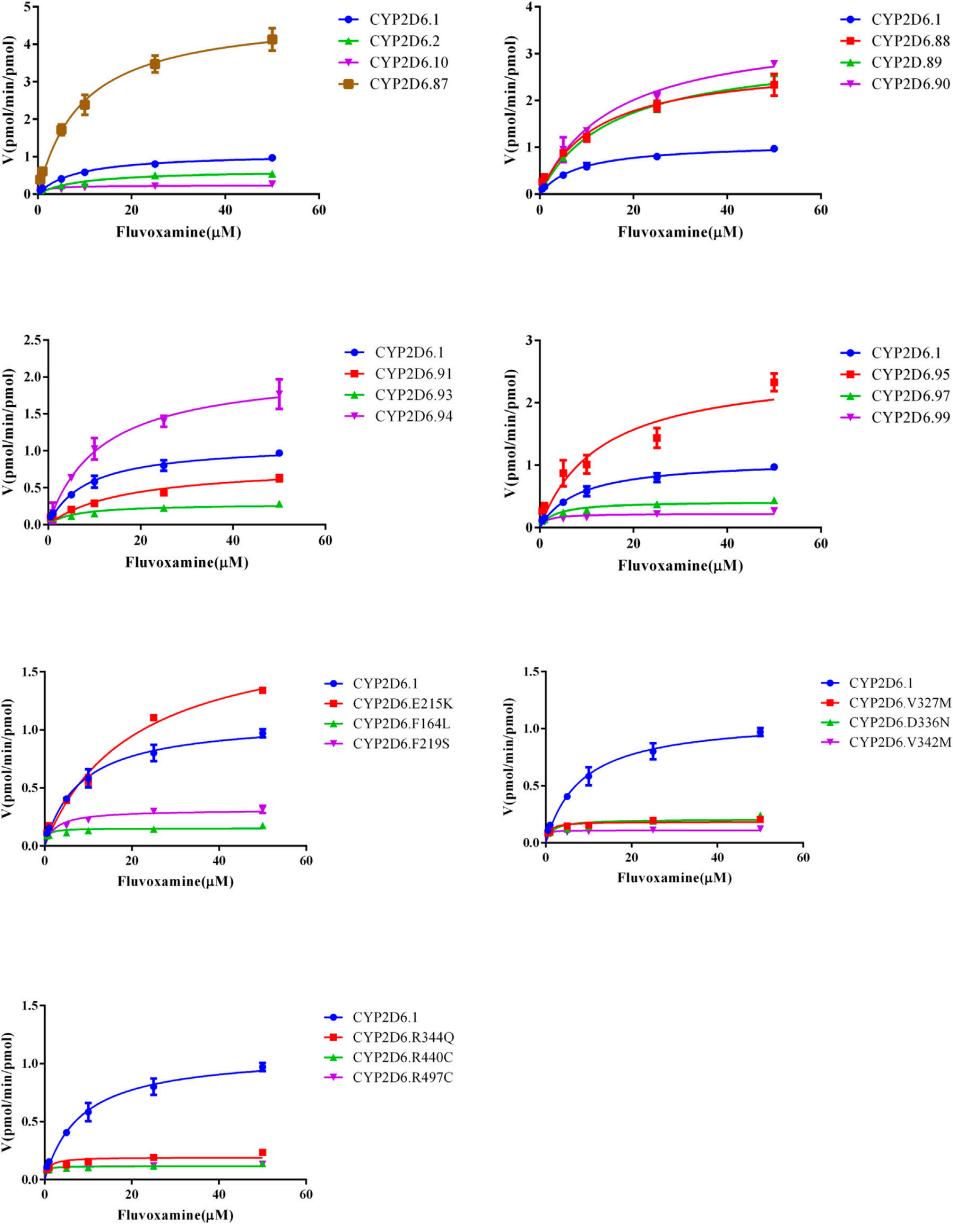
Michaelis–Menten curves of CYP2D6 in disposition of fluvoxamine. The reaction was performed as indicated in the section of method, *n* = 3.

Michaelis curves and kinetic parameters of CYP2D6.1 and other variants in metabolizing fluvoxamine were shown in Figure 2 and Table 1, respectively. Based on maximum reaction velocity (V_max_), they can be divided into four groups. No significant differences were observed between CYP2D6.1 and CYP2D.2, V104, 90, C161S, D337G, E215K, R497C. Among them, CYP2D6.92 and 96 almost lost enzymatic function. Moreover, the V_max_ of CYP2D6.A5V, 89 and 95 increased remarkably, ranging from 270.31% to 441.17%. In opposite, the remaining variants decreased significantly, ranging from 9.93% to 39.25%. From the michaelis constant (K_m_), a lot of them decreased obviously compared with CYP2D6.1, including CYP2D6.10, 97, R88P, F164L, F219S, V327M, D336N, V342M, R344Q, R440C, R497C. Besides, the other variants had no significant difference. Finally, the intrinsic clearance (Clint) and relative clearance were determined. In all, activities of seven variants, involving CYP2D6.A5V, V104A, D337G, F164L, V342M, R440C, R497C, increased compared with CYP2D6.1, ranging from 153.63% to 394.31%. The others’ The remaining variants showed no statistical difference in intrinsic clearance. In addition, CYP2D6.92 and 96 had no significant activity. The rest of the variants had different effect of reduced metabolic activity.

**TABLE 1:** Kinetic parameters of fluvoxamine catalyzing in CYP2D6.

CYP	*V*_*max*_ (*pmol/min/pmol*)	*K*_*m*_ (*pmol*)	*CL (μL/min/pmol)*	%
2D6.1	1.098 ± 0.015	8.415 ± 0.797	0.131 ± 0.012	100.00 ± 9.27
2D6.2	0.652 ± 0.093	9.636 ± 4.023	0.073 ± 0.018	55.254 ± 13.364
2D6.10	0.233 ± 0.001*	1.751 ± 0.169*	0.134 ± 0.013	101.865 ± 9.613
2D6.A5V	4.844 ± 0.434*	9.438 ± 1.358	0.516 ± 0.034*	393.295 ± 25.911*
2D6.V104A	2.879 ± 0.237	12.523 ± 1.792	0.231 ± 0.015*	176.140 ± 11.160*
2D6.89	3.096 ± 0.223*	15.627 ± 1.793	0.199 ± 0.011	151.434 ± 8.154
2D6.90	3.627 ± 0.433	16.373 ± 5.954	0.236 ± 0.059	179.493 ± 45.215
2D6.C161S	0.816 ± 0.110	16.233 ± 2.404	0.050 ± 0.001	38.336 ± 0.864
2D6.92	N.D.	N.D.	N.D.	N.D.
2D6.93	0.259 ± 0.002*	4.058 ± 0.369	0.064 ± 0.006	48.790 ± 4.378
2D6.D337G	2.142 ± 0.187	10.645 ± 1.196	0.202 ± 0.009*	153.626 ± 6.718*
2D6.R388H	2.968 ± 0.109*	18.053 ± 2.090	0.166 ± 0.015	126.083 ± 11.685
2D6.96	N.D.	N.D.	N.D.	N.D.
2D6.97	0.431 ± 0.007*	3.483 ± 0.652*	0.127 ± 0.024	96.463 ± 18.420
2D6. R88P	0.234 ± 0.001*	1.791 ± 0.230*	0.132 ± 0.018	100.802 ± 13.791
2D6.F164L	0.147 ± 0.008*	0.514 ± 0.053*	0.287 ± 0.016*	218.589 ± 12.179*
2D6.E215K	1.723 ± 0.219	17.960 ± 3.975	0.098 ± 0.012	74.351 ± 9.147
2D6.F219S	0.349 ± 0.008*	3.749 ± 0.245*	0.093 ± 0.008	71.168 ± 5.992
2D6.V327M	0.199 ± 0.020*	0.981 ± 0.222*	0.207 ± 0.028	157.504 ± 21.434
2D6.D336N	0.202 ± 0.006*	1.532 ± 0.215*	0.134 ± 0.017	101.831 ± 12.972
2D6.V342M	0.109 ± 0.002*	0.212 ± 0.022*	0.518 ± 0.044*	394.310 ± 33.268*
2D6.R344Q	0.203 ± 0.002*	1.270 ± 0.194*	0.162 ± 0.024	123.621 ± 18.515
2D6.R440C	0.117 ± 0.001*	0.282 ± 0.013*	0.416 ± 0.018*	317.234 ± 14.091*
2D6.R497C	0.116 ± 0.0018	0.266 ± 0.014*	0.436 ± 0.021*	331.993 ± 15.802*

*N* = 3, variants vs. CYP2D6.1.

*p < 0.05.

### Effects of apatinib on fluvoxamine metabolism in rats

**FIGURE 3:**
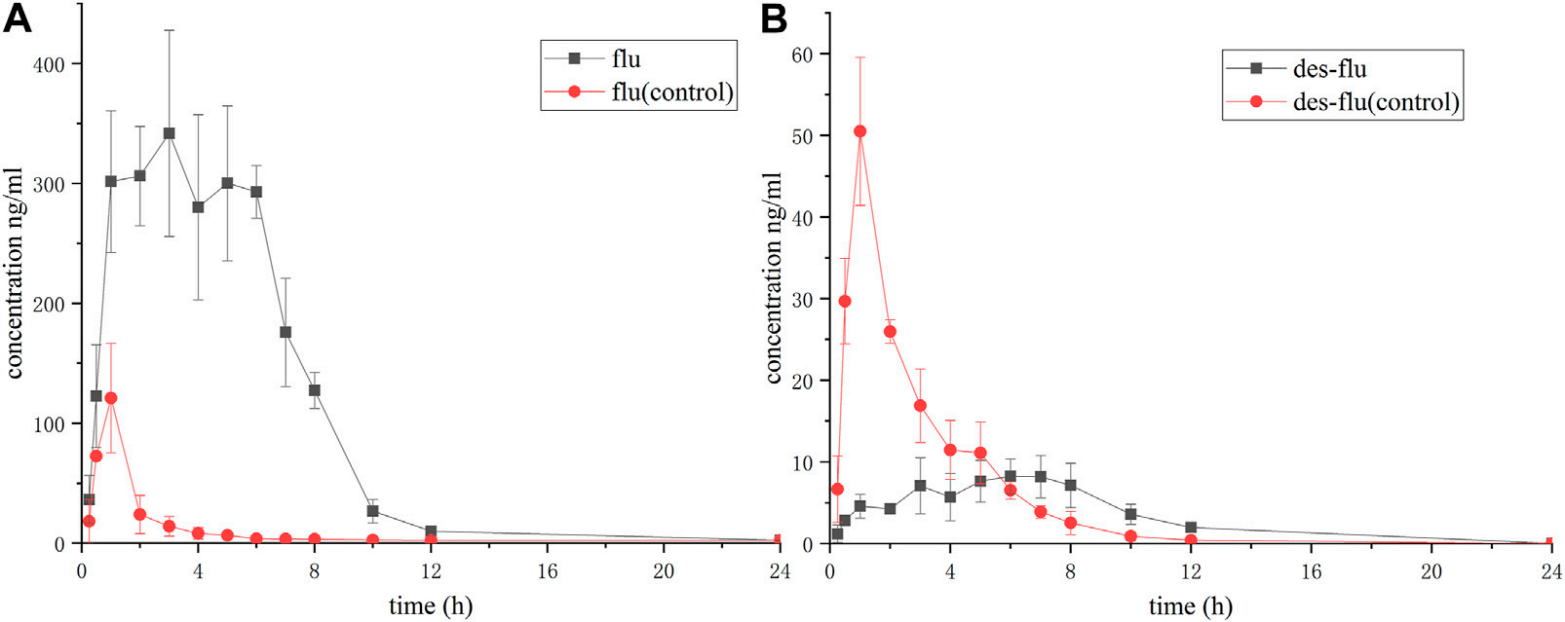
Concentration-time curve of fluvoxamine and its metabolite. The rats were administrated with fluvoxamine via gavage. As follows, the tail vein blood was collected, and was subjected to UPLC-MS/MS assay. **(A)** Desmethyl fluvoxamine, **(B)** Fluvoxamine. The curve was plotted using Prism 5, *n* = 6.

As Figure 3A showed, when co-administration of fluvoxamine with apatinib, the production of desmethyl fluvoxamine was inhibited dramatically. The peak of the Y-axis of the time-concentration curve decreased, and the curve shifted to the right with significantly enhanced in T_max_. The AUC, t_1/2_, and C_max_ reduced, but no significant difference was found, Table 2. Accordingly, the concentration of fluvoxamine increased remarkably after combination, Figure 3B. The AUC_(0-t)_, AUC_(0-∞)_ and C_max_ values of fluvoxamine increased by 8.52-, 2.78-, 2.90-time, respectively. In addition, T_max_ is prolonged by 3.80 times, CLz/F is reduced by about one time, and t_1/2z_ is reduced by nearly one time, Table 3.

**TABLE 2:** Pharmacokinetic parameters of desmethyl fluvoxamine.

Pharmacokinetic parameters	Unit	Group B	Group A
AUC(0-t)	µg/L*h	80.103 ± 18.294	113.125 ± 37.015
AUC(0-∞)	µg/L*h	80.178 ± 18.307	113.246 ± 37.062
MRT (0-t)	h	6.781 ± 1.005*	3.446 ± 0.578
MRT (0-∞)	h	6.8 ± 1.01*	3.473 ± 0.578
t1/2z	h	1.9 ± 0.224	2.525 ± 0.793
T_max_	h	5.333 ± 2.251*	1.803 ± 0.492
CLz/F	L/h/kg	131.088 ± 34.451	99.297 ± 41.451
Vz/F	L/kg	356.789 ± 85.786	358.178 ± 217.799
C_max_	µg/L	10.665 ± 3.71*	34.263 ± 19.449

AUC, area under curve; MRT, mean retention time; t_1/2z_, elimination half time; T_max_, peak time; Vz/F, apparent volume of distribution; CLz/F, blood clearance; C_max_, maximum blood concentration. Group B vs. Group A.

*p < 0.05, *n* = 6.

**TABLE 3:** Pharmacokinetic parameters of fluvoxamine.

Pharmacokinetic parameters	Unit	Group B	Group A
AUC_(0-t)_	µg/L*h	1,723.097 ± 602.157*	180.900 ± 100.454
AUC_(0-∞)_	µg/L*h	1,724.820 ± 600.410*	456.181 ± 163.803
MRT_(0-t)_	h	5.191 ± 0.673	6.095 ± 2.380
MRT_(0-∞)_	h	5.225 ± 0.659*	92.049 ± 44.632
t_1/2 z_	h	1.637 ± 0.961*	2.145 ± 37.094
T_max_	h	4.000 ± 1.549*	0.833 ± 0.258
CLz/F	L/h/kg	6.396 ± 2.110*	24.166 ± 7.713
Vz/F	L/kg	16.228 ± 13.475*	2,618.204 ± 879.13
C_max_	µg/L	274.989 ± 100.275*	70.595 ± 62.901

Group B vs. Group A.

*p < 0.05, *n* = 6.

### The effect of apatinib on fluvoxamine metabolism *in vitro*

**FIGURE 4:**
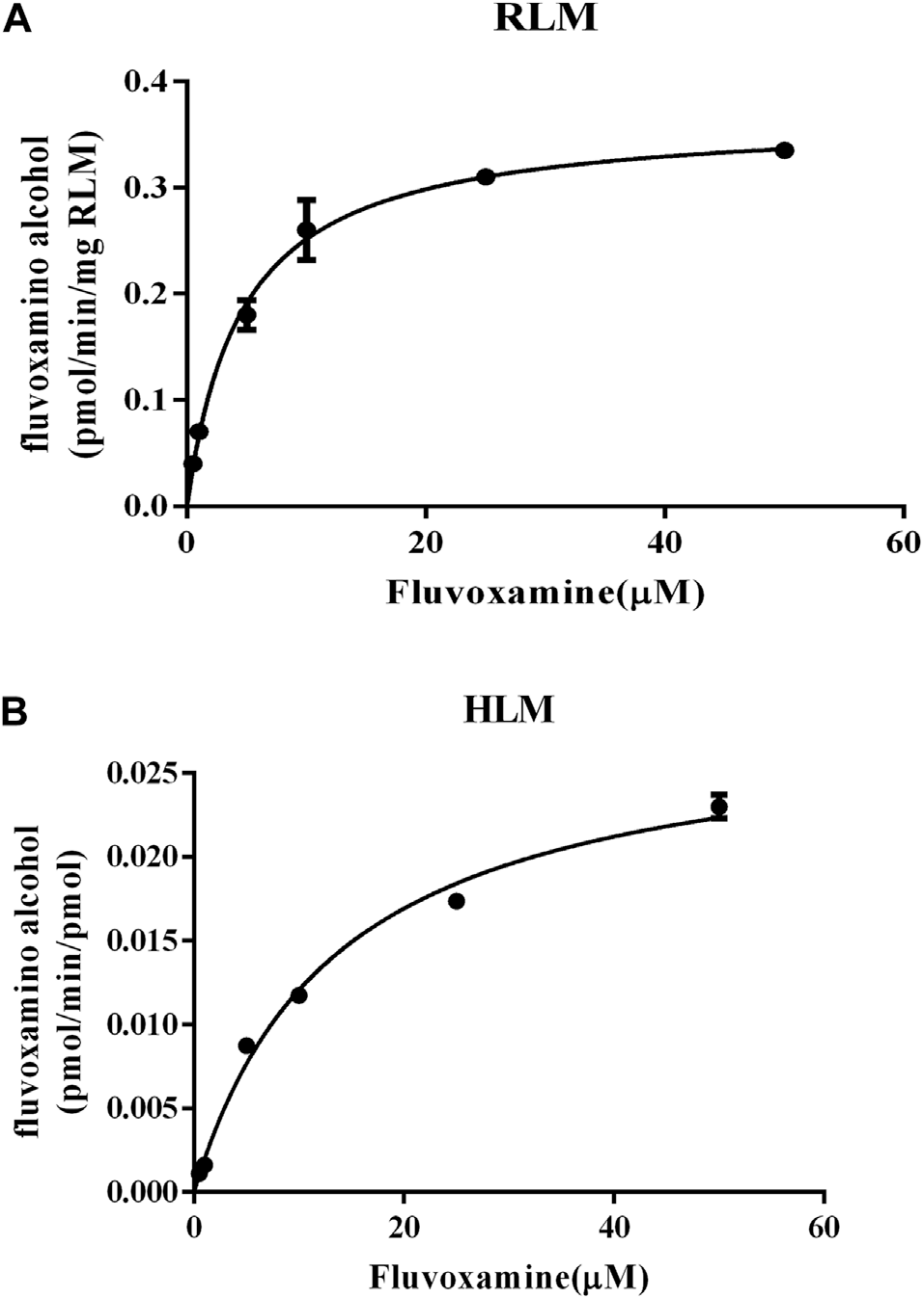
Michaelis–Menten curves of fluvoxamine metabolism in microsomes. **(A)** RLM. **(B)** HLM. *n* = 3.

**FIGURE 5:**
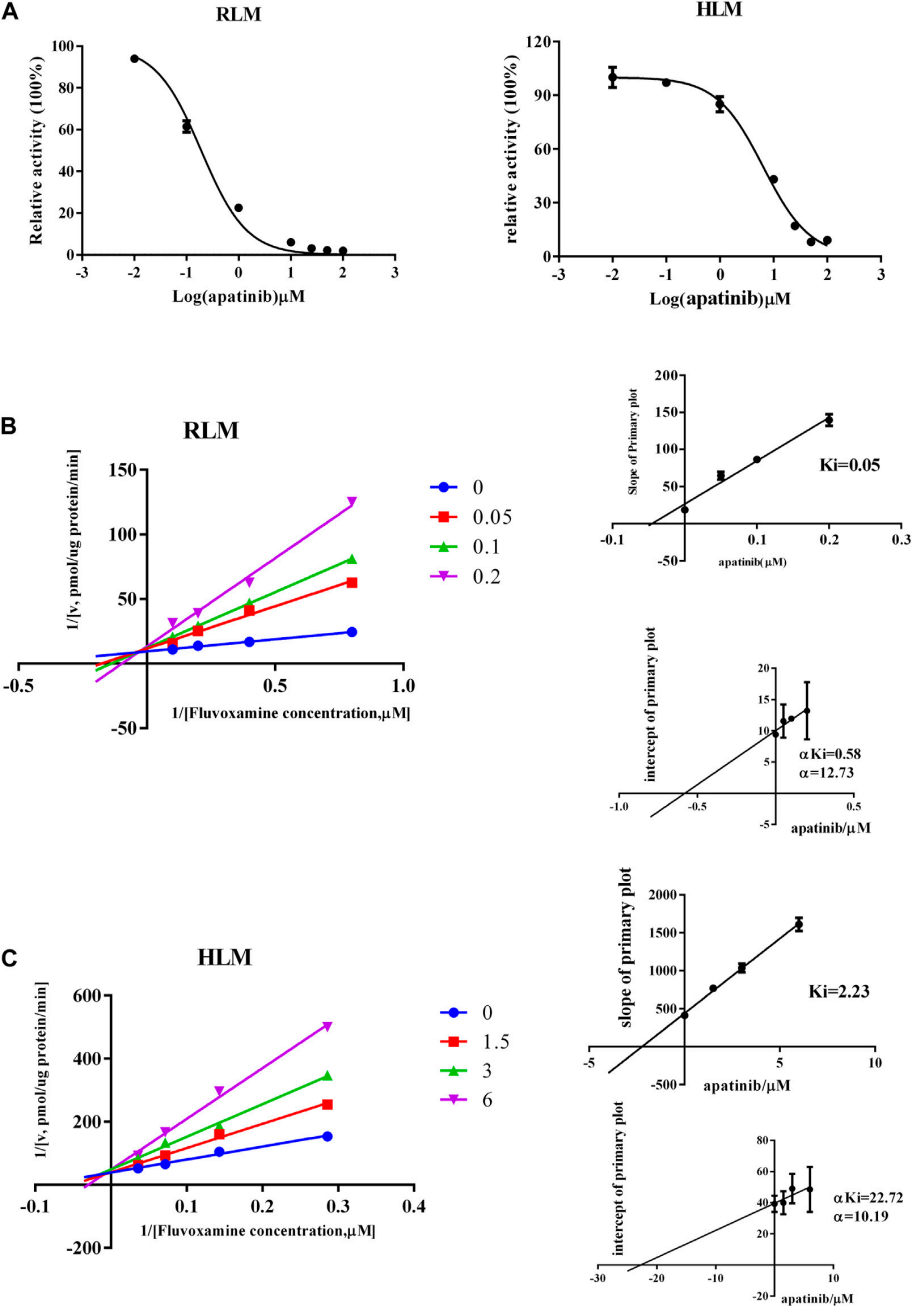
Apatinib inhibited the metabolism of fluvoxamine with a mixed mechanism both in RLM and HLM. **(A)** The effect of apatinib on inhibiting fluvoxamine metabolism. **(B,C)** Lineweaver-Burk plot and the secondary plot for Ki in the inhibition of fluvoxamine catalyzing, *n* = 3.

**FIGURE 6:**
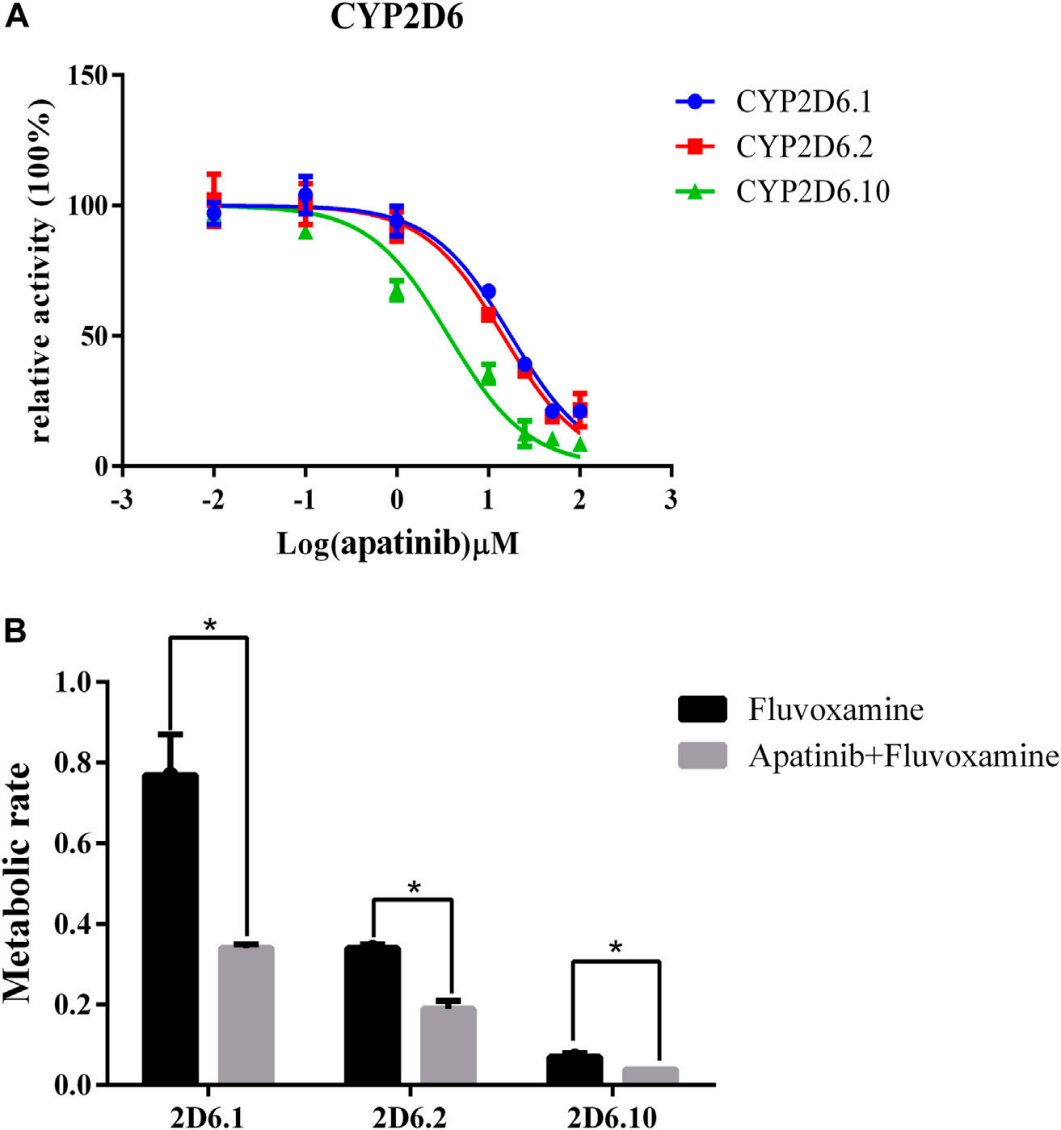
The different effect of apatinib on fluvoxamine metabolism in three CYP2D6 variants. **(A)** The catalytic profile of fluvoxamine in CYP2D6.1, 2 and 10. **(B)** The effect of apatinib on the metabolic rate of fluvoxamine in CYP2D6.1, 2, and 10, *n* = 3, *p < 0.05.

To study the mechanism underlied drug-drug interaction, the enzymatic reaction was performed using RLM, HLM. As shown in Figure 4, the K_m_ of fluvoxamine metabolizing was 4.738 μM in RLM and 13.54 μM in HLM. To evaluate the inhibitory potency, the IC_50_ was determined. Fluvoxamine was dose-dependently inhibited by apatinib in RLM with IC_50_ of 0.19 μM, Figure 5A. In HLM, it’s 6.419 μM, Figure 5A. Mechanistically, apatinib inhibited fluvoxamine metabolism with a mixed manner in the RLM. The Ki is 0.05 μM, Figure 5B. Meanwhile, it is the same in HLM with Ki of 2.23 μM, Figure 5C. To further investigate differences in inhibitory activity among different CYP2D6 variants, IC_50_ was determined using CYP2D6.1, CYP2D6.2 and CYP2D6.10. It’s 17.58, 14.46 and 3.673 μM accordingly, Figure 6A. The relative metabolic rates of fluvoxamine were 44.32%, 54.03%, and 57.69%, respectively.

## Discussion

CYP2D6 accounts for about 2% of total CYP abundance in liver, and catalyzing metabolism of 20%–30% therapeutic drugs (Zhou et al., 2016; Taylor et al., 2020). To date, 149 CYP2D6 alleles have been reported (LLerena et al., 2014). However, the activities of a vast majority of variants are still unclear. Therefore, elucidating the effect of mutation on enzyme activity will help to understand the metabolic characteristics of CYP2D6 substrate drugs. Moreover, it will provide the basic data for precise medicine.

Fluvoxamine is almost completely absorbed through the gastrointestinal tract, and is mainly metabolized into desmethyl fluvoxamine in the liver through CYP2D6 and CYP1A2 pathway (van Harten, 1995). Interestingly, CYP2D6 is rarely being induced, but has abundance gene polymorphism. Therefore, genetic polymorphisms of CYP2D6 are likely to cause differences in plasma exposure of fluvoxamine. This study demonstrated that CYP2D6.A5V, V104A, D337G, F164L, V342M, R440C, R497C showed higher catalytic activity compared with CYP2D6.1. The patients carry these mutations would probably be sub-therapied. In contrast, CYP2D6*92 and *96 almost lost function in catalyzing fluvoxamine. CYP2D6*10 is predominantly distributed in East-Asia (Lin et al., 2016). It has been reported that its activity is significantly reduced. In the present study, we found that the V_max_ of CYP2D6 decreased, the K_m_ was also decreased accordingly. Therefore, the relative clearance is nearly the equal to CYP2D6.1. We think CYP2D6 has a certain selectivity for substrate drugs (Ingelman-Sundberg, 2005). The structure of the compound and the affinity of compound to the enzyme determine the characteristics of reaction (Zhou et al., 2009). However, these speculations require further study. Taken together, our data suggest that CYP2D6 gene polymorphisms indeed have varying degrees of impact on fluvoxamine metabolism.

Due to the existence of various complications, cancer patients usually take multiple drugs in combination, which can easily lead to drug-drug interaction (Flepisi et al., 2014; Moghaddas et al., 2021). Identifying potential drug interactions will help guide rational drug use and improve patients’ quality of life. Anxiety is a common complication in cancer patients (Kapfhammer, 2015). Therefore, combining apatinib with fluvoxamine is an effective dosing strategy. Many studies had shown that apatinib interacts with antipsychotics like buspirone and venlafaxine (Bao et al., 2018; Zhang et al., 2020). In this study, the data demonstrated that it can also inhibit the metabolizing of fluvoxamine via suppression the activities of microsomes system, especially CYP2D6. This inhibitory effect was similar even with different CYP2D6 alleles. Although this study preliminarily demonstrated differences in the rates of mutants metabolizing fluvoxamine through *in vitro* experiments, the affinity of the substrate to the enzyme was not determined, as well as *in vivo* experiments. Therefore, further data cannot be used to explain the *in vivo* situation, which has certain limitations in guiding clinical drug treatment. Since fluvoxamine and apatinib may be used clinically in combination, in this study we combined *in vivo* and *in vitro* experiments to demonstrate the interaction between fluvoxamine and apatinib. At the same time, the relevant experiments of HLM *in vitro* confirmed that apatinib may have a certain inhibitory effect on fluvoxamine in humans. In all, the present study provides basic data for the clinical application of fluvoxamine, especially in cancer patients. This prescription needs to be vigilant and prevent the occurrence of adverse reactions. Although prolonging lifespan is the primary goal for cancer patients, maximizing the quality of life is an urgent clinical problem, and this study provides limited data support for this goal.

## Data Availability

The original contributions presented in the study are included in the article/Supplementary Material, further inquiries can be directed to the corresponding authors.
