# Supplementary material for: CYP2D6 gene polymorphism and apatinib affect the metabolic profile of fluvoxamine
Source: Front Pharmacol. 2022 Sep 2;13:985159. doi: 10.3389/fphar.2022.985159 (PMC9479105; doi:10.3389/fphar.2022.985159)
Supplement: Supplementary file 1 [file DataSheet1.docx]

***Supplementary information***

**CYP2D6 gene polymorphism and apatinib affect the metabolic profile of fluvoxamine**

Supplementary Figures: 1

Supplementary Tables: 9


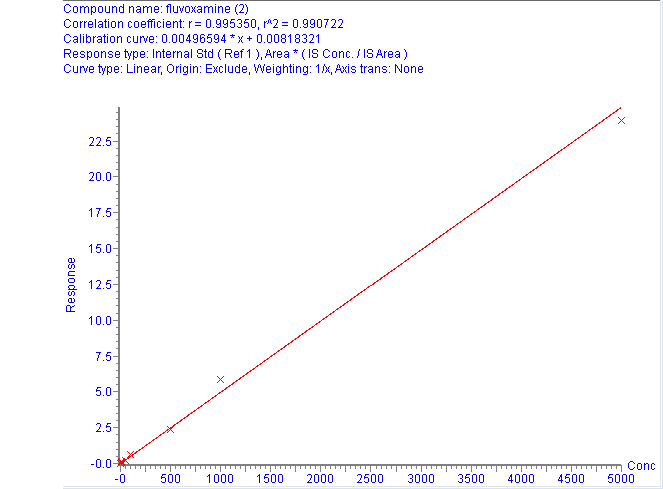

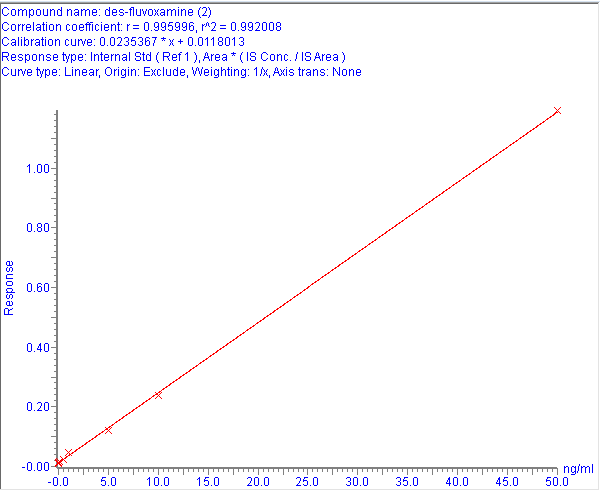


1. A
2. B
3. A

**Supplementary Figure S1.**The standard curve of Fluvoxamine（A）and desmethyl Fluvoxamine （B）.

| Labeled concentration (ng/mL) | Detection concentration (ng/mL) | RSD (%) | RE (%) |
| --- | --- | --- | --- |
| 2 | 2.253 ± 0.165 | 7.30 | 12.63 |
| 2000 | 2192.629 ± 71.582 | 3.26 | 9.63 |
| 4000 | 4074.252 ± 224.319 | 5.51 | 1.86 |

**Supplementary Table S1.** Intra-day Precision and accuracy of Fluvoxamine in rats plasma (n=6).

**Supplementary Table S2.** Intra-day Precision and accuracy of desmethyl Fluvoxamine in rats plasma (n=6).

| Labeled concentration (ng/mL) | Detection concentration (ng/mL) | RSD (%) | RE (%) |
| --- | --- | --- | --- |
| 0.02 | 0.021 ± 0.002 | 9.44 | 3.71 |
| 20 | 20.351 ± 0.367 | 1.80 | 1.76 |
| 40 | 38.412 ± 0.251 | 0.65 | -3.97 |

**Supplementary Table S3.** Inter-day Precision and accuracy of Fluvoxamine in rats plasma (n=6).

| Labeled concentration (ng/mL) | Detection concentration (ng/mL) | RSD (%) | RE (%) |
| --- | --- | --- | --- |
| 2 | 1.825 ± 0.127 | 6.96 | -8.76 |
| 2000 | 2135.444 ± 120.048 | 5.62 | 6.77 |
| 4000 | 4550.651 ± 350.688 | 7.71 | 13.77 |

**Supplementary Table S4.** Inter-day Precision and accuracy of desmethyl Fluvoxamine in rats plasma (n=6).

| Labeled concentration (ng/mL) | Detection concentration (ng/mL) | RSD (%) | RE (%) |
| --- | --- | --- | --- |
| 0.02 | 0.020 ± 0.002 | 8.06 | -0.01 |
| 20 | 17.329 ± 0.536 | 3.10 | -13.35 |
| 40 | 37.144 ± 1.982 | 5.34 | -7.14 |

**Supplementary Table S5.** Recovery and matrix effect of Fluvoxamine (n=6).

| Labeled concentration (ng/mL) | Recovery rate (%) | Matrix effect (%) |
| --- | --- | --- |
| 2 | 96 | 103 |
| 2000 | 99 | 106 |
| 4000 | 96 | 103 |

**Supplementary Table** **S6**. Recovery and matrix effect of desmethyl Fluvoxamine in rats plasma (n=6).

| Labeled concentration (ng/mL) | Recovery rate (%) | Matrix effect (%) |
| --- | --- | --- |
| 0.02 | 108.57 | 87.14 |
| 20 | 111.41 | 87.29 |
| 40 | 86.36 | 99.60 |

**Supplementary Table S7**. Stability of fluvoxamine plasma quality control samples in different environments (n=6)

| environment | Labeled concentration  (ng/mL) | Detection concentration  (ng/mL) | RSD (%) | RE (%) |
| --- | --- | --- | --- | --- |
| Room temperature, 6 hours | 2 | 2.21 ± 0.30 | 13.56 | -7 |
|  | 2000 | 1992.80 ±43.54 | 2.18 | 1 |
|  | 4000 | 3980.81 ± 172.14 | 4.32 | 5 |
| -80℃, 30 days | 2 | 1.83 ± 0.19 | 10.24 | 0 |
|  | 2000 | 1993.37 ± 58.92 | 2.96 | 4 |
|  | 4000 | 4016.85 ± 144.39 | 3.59 | 0 |
| 4℃，24 hours | 2 | 1.85 ± 0.23 | 12.48 | 11 |
|  | 2000 | 2026.52 ± 118.71 | 5.86 | -5 |
|  | 4000 | 4062.80 ± 210.02 | 5.17 | -3 |
| Freeze-thaw cycle | 2 | 0.02 ± 0.00 | 12.20 | 10 |
|  | 2000 | 19.37 ± 0.73 | 3.75 | -3.17 |
|  | 4000 | 38.57 ± 0.68 | 1.76 | -3.57 |

**Supplementary Table S8**. Stability of desmethyl Fluvoxamine plasma quality control samples in different environments (n=6)

| environment | Labeled concentration  (ng/mL) | Detection concentration  (ng/mL) | RSD (%) | RE (%) |
| --- | --- | --- | --- | --- |
| Room temperature, 6 hours | 0.02 | 0.02 ±0.00 | 10.07 | 1 |
|  | 20 | 18.59 ± 0.30 | 1.63 | -7.04 |
|  | 40 | 37.80 ± 0.73 | 1.93 | -5.51 |
| -80℃, 30 days | 0.02 | 0.02 ± 0.00 | 14.82 | 4 |
|  | 20 | 21.71 ± 0.73 | 3.36 | 8.57 |
|  | 40 | 41.73 ± 0.70 | 1.67 | 4.32 |
| 4℃，24 hours | 0.02 | 0.02 ± 0.00 | 12.20 | 10 |
|  | 20 | 19.37 ± 0.73 | 3.75 | -3.17 |
|  | 40 | 38.57 ± 0.68 | 1.76 | -3.57 |
| Freeze-thaw cycle | 0.02 | 0.02 ± 0.00 | 4.23 | -1 |
|  | 20 | 22.24 ± 1.11 | 5.01 | 11.18 |
|  | 40 | 41.14 ± 1.57 | 3.81 | 2.84 |

**Supplementary Table S9**.The IC50 values and inhibitory effects of Apatinib on Fluvoxamine metabolism in RLM, HLM and CYP2d6.1,2,10.

|  | IC_50_ values (μM) | Inhibition type | K_i_ (μM) | αK_i_ (μM) | α |
| --- | --- | --- | --- | --- | --- |
| RLM | 0.19 | mixed | 0.05 | 0.58 | 12.73 |
| HLM | 6.419 | mixed | 2.23 | 22.72 | 10.19 |
| CYP2D6.1 | 17.58 | — | — | — | — |
| CYP2D6.2 | 14.46 | — | — | — | — |
| CYP2D6.10 | 3.673 | — | — | — | — |
